# Increasing risk-concordant cardiovascular care in diverse health systems: a mixed methods pragmatic stepped wedge cluster randomized implementation trial of shared decision making (SDM4IP)

**DOI:** 10.1186/s43058-021-00145-6

**Published:** 2021-04-21

**Authors:** Jennifer L. Ridgeway, Megan E. Branda, Derek Gravholt, Juan P. Brito, Ian G. Hargraves, Sandra A. Hartasanchez, Aaron L. Leppin, Yvonne L. Gomez, Devin M. Mann, Vivek Nautiyal, Randal J. Thomas, Emma M. Behnken, Victor D. Torres Roldan, Nilay D. Shah, Charanjit S. Khurana, Victor M. Montori

**Affiliations:** 1 Robert D. and Patricia E. Kern Center for the Science of Health Care Delivery, Mayo Clinic, 200 First Street SW, Rochester, MN 55905 USA; 2 Knowledge and Evaluation Research Unit, Mayo Clinic, 200 First Street SW, Rochester, MN 55905 USA; 3 Department of Biostatistics and Informatics, Colorado School of Public Health, University of Colorado-Denver Anschutz Medical Campus, 13001 East 17th Place, 3rd Floor, Mail Stop B119, Aurora, CO 80045 USA; 4 Division of Diabetes, Endocrinology, Metabolism, and Nutrition, Department of Medicine, Mayo Clinic, 200 First Street SW, Rochester, MN 55905 USA; 5 Altru Health System, 1380 S. Columbia Road, Grand Forks, ND 58206 USA; 6 Department of Population Health, NYU Grossman School of Medicine, 530 1st Avenue, New York, NY 10016 USA; 7 Wellstar Cardiovascular Medicine, 55 Whitcher Street, NE, Suite 350, Marietta, GA 30060 USA; 8 Division of Preventive Cardiology, Department of Cardiovascular Medicine, Mayo Clinic, 200 First Street SW, Rochester, MN 55905 USA; 9 Virginia Hospital Center Physician Group-Cardiology, 1715 North George Mason Drive, Arlington, VA 22205 USA

**Keywords:** Shared decision making, Risk-treatment paradox, Cardiovascular treatment, Implementation science, Mixed methods, Implementation facilitation

## Abstract

**Background:**

The primary prevention of cardiovascular (CV) events is often less intense in persons at higher CV risk and vice versa. Clinical practice guidelines recommend that clinicians and patients use shared decision making (SDM) to arrive at an effective and feasible prevention plan that is congruent with each person’s CV risk and informed preferences. However, SDM does not routinely happen in practice. This study aims to integrate into routine care an SDM decision tool (CV Prevention Choice) at three diverse healthcare systems in the USA and study strategies that foster its adoption and routine use.

**Methods:**

This is a mixed method, hybrid type III stepped wedge cluster randomized study to estimate (a) the effectiveness of implementation strategies on SDM uptake and utilization and (b) the extent to which SDM results in prevention plans that are risk-congruent. Formative evaluation methods, including clinician and stakeholder interviews and surveys, will identify factors likely to impact feasibility, acceptability, and adoption of CV Prevention Choice as well as normalization of CV Prevention Choice in routine care. Implementation facilitation will be used to tailor implementation strategies to local needs, and implementation strategies will be systematically adjusted and tracked for assessment and refinement. Electronic health record data will be used to assess implementation and effectiveness outcomes, including CV Prevention Choice reach, adoption, implementation, maintenance, and effectiveness (measured as risk-concordant care plans). A sample of video-recorded clinical encounters and patient surveys will be used to assess fidelity. The study employs three theoretical approaches: a determinant framework that calls attention to categories of factors that may foster or inhibit implementation outcomes (the Consolidated Framework for Implementation Research), an implementation theory that guides explanation or understanding of causal influences on implementation outcomes (Normalization Process Theory), and an evaluation framework (RE-AIM).

**Discussion:**

By the project’s end, we expect to have (a) identified the most effective implementation strategies to embed SDM in routine practice and (b) estimated the effectiveness of SDM to achieve feasible and risk-concordant CV prevention in primary care.

**Trial registration:**

ClinicalTrials.gov, NCT04450914. Posted June 30, 2020

**Trial status:**

This study received ethics approval on April 17, 2020. The current trial protocol is version 2 (approved February 17, 2021). The first subject had not yet been enrolled at the time of submission.

Contributions to the literature

- Shared decision making (SDM) may mitigate the cardiovascular risk-treatment paradox, whereby most clinical interventions are implemented in people at lower risk, and few among people at high risk, but SDM implementation has faltered in practice.
- This study will integrate an SDM tool into the electronic record workflow of three diverse health systems, engage stakeholders in tailoring implementation strategies, and iteratively track, assess, and refine them.
- The hybrid stepped-wedge cluster-randomized trial will use a robust theoretical approach and mixed methods to identify implementation strategies that foster routine, high-quality SDM and estimate the effectiveness of SDM to achieve feasible, risk-concordant cardiovascular prevention.

## Background

Cardiovascular (CV) disease is a leading cause of premature mortality in the USA [1], accounting for 1 of every 3 deaths, of morbidity, and of healthcare expenditures [2, 3]. While public health interventions (e.g., smoking bans) are best suited to reduce the burden of CV disease in the population, clinical prevention can more precisely target high-risk individuals. Development of tools to estimate an individual’s CV risk and clinical interventions to reduce that risk support an increasingly targeted approach [4], which should translate into a substantial reduction in CV burden. Yet, a persistent finding in real-world evidence indicates most clinical interventions are implemented in people at relatively lower CV risk, and few among people at the highest risk [5, 6]. This so-called risk-treatment paradox has been observed repeatedly across a range of CV-risk reducing interventions such as statins and aspirin [5–7]. Among patients with type 2 diabetes, research has found that treatments to reduce CV risk, including SGLT2 inhibitors and GLP1 receptor agonists, are less likely to be prescribed to patients at the highest CV risk [8] despite trial data showing benefits in high-risk groups. Black patients (vs. white), women (vs. men), and those covered by Medicare Advantage (vs. commercial insurance with identical formulary coverage) have also been found to be less likely to receive these agents [8, 9].

Clinicians’ failure to document or consider comorbidities may contribute to paradoxical treatment patterns. For example, clinicians may not be aware of or address psychosocial risks (e.g., undiagnosed depression, functional capacity) that would otherwise inform appropriate preventive strategies [6]. Further complicating the matter, patients at risk of CV disease are often unaware of their own risk, or they have difficulty assessing the relative benefits of risk-reduction strategies [10]. Patients may seek to understand these benefits and risks before commencing treatment [11], but clinicians do not always provide this information. Even patients who have implemented preventive therapies are sometimes unable to connect the medications they are taking with their preventive goal [12].

When patients and clinicians work together, they can consider the specific threat posed by CV disease and craft a sensible and feasible plan of care that also considers the patient’s concerns. This process of shared decision making (SDM) can mitigate the risk-treatment paradox by reducing risk blindness and improving the fit between the patient’s situation and the potential benefits, harms, and burden of implementing the preventive regimen [13–15]. The appropriate treatment regimen should be commensurate to each person’s level of CV risk and should make intellectual, practical, and emotional sense for each patient [16].

The 2019 American College of Cardiology/American Heart Association (ACC/AHA) guidelines strongly (Class I) recommended SDM for primary CV prevention, although they provide limited guidance on implementing this recommendation [4]. Risk estimation and risk communication tools, including SDM tools, can help facilitate SDM during the clinical encounter. Tools like these, sometimes called encounter or conversation decision aids in contrast to patient decision aids [17], have proven effective in improving patient experience by increasing health-related knowledge and reducing decisional conflict, while potentially improving clinician satisfaction with medical decision making [18, 19]. However, broad uptake of SDM has faltered in practice. Barriers to SDM adoption include patient and clinician attitudes or preferences, perceived time constraints on clinic visits, concerns about reduced uptake of preventive interventions with SDM [20], and organizational or public policies that favor traditional disease management approaches, e.g., the pursuit of population targets [21].

Integration of point-of-care SDM tools into the electronic health record (EHR) and associated workflows may support SDM but be insufficient for normalizing SDM into clinical routines [22–24]. Likewise, the availability of an SDM tool does not dictate fidelity to an SDM approach [25]. Implementation strategies are the actions or methods aimed at enhancing adoption of evidence-based practices [26]. Development of targeted, multilevel implementation strategies may effectively address barriers to SDM adoption, including behavioral aspects such as clinician motivation to use SDM [27].

### Study objectives

The overall goal of this study is to identify implementation approaches that foster routine, high-quality SDM about CV prevention in people cared for in primary preventive care settings. There are two specific aims: (1) To determine the effectiveness of implementation strategies on SDM uptake and routine use by employing a multilevel evaluation of (1a) the practice context and methods to engage users in identifying and tailoring implementation strategies, (1b) the implementation outcomes (e.g., reach, adoption) associated with tailored implementation strategies, and (1c) the work clinicians do to normalize use of the SDM tool in routine practice; and (2) To estimate the extent to which SDM, as implemented in usual primary care settings, results in individual CV prevention plans that are evidence-based and congruent with each person’s estimated CV risk.

## Methods/design

### SDM intervention

The SDM tool employed in this trial—CV Prevention Choice—is based on Statin Choice, a point-of-care SDM tool designed in 2005 and evaluated in three RCTs [28–30]. After estimating the 10-year risk of atherosclerotic cardiovascular disease (ASCVD), the tool presents a 100-person pictograph of this risk alongside of the reduced risk achieved were the patient to use standard or high-dose statins, obtained by applying the relative risk reduction estimated in large scale efficacy RCTs. The tool also supports conversations about statins’ other desirable and adverse effects, as well as details about how they are taken and their out-of-pocket costs. This phase of the discussion ensures that the evidence-based preventive strategy under consideration makes sense, is consistent with the values and preferences for prevention of each patient, and is feasible for that patient [30].

**Fig. 1:**
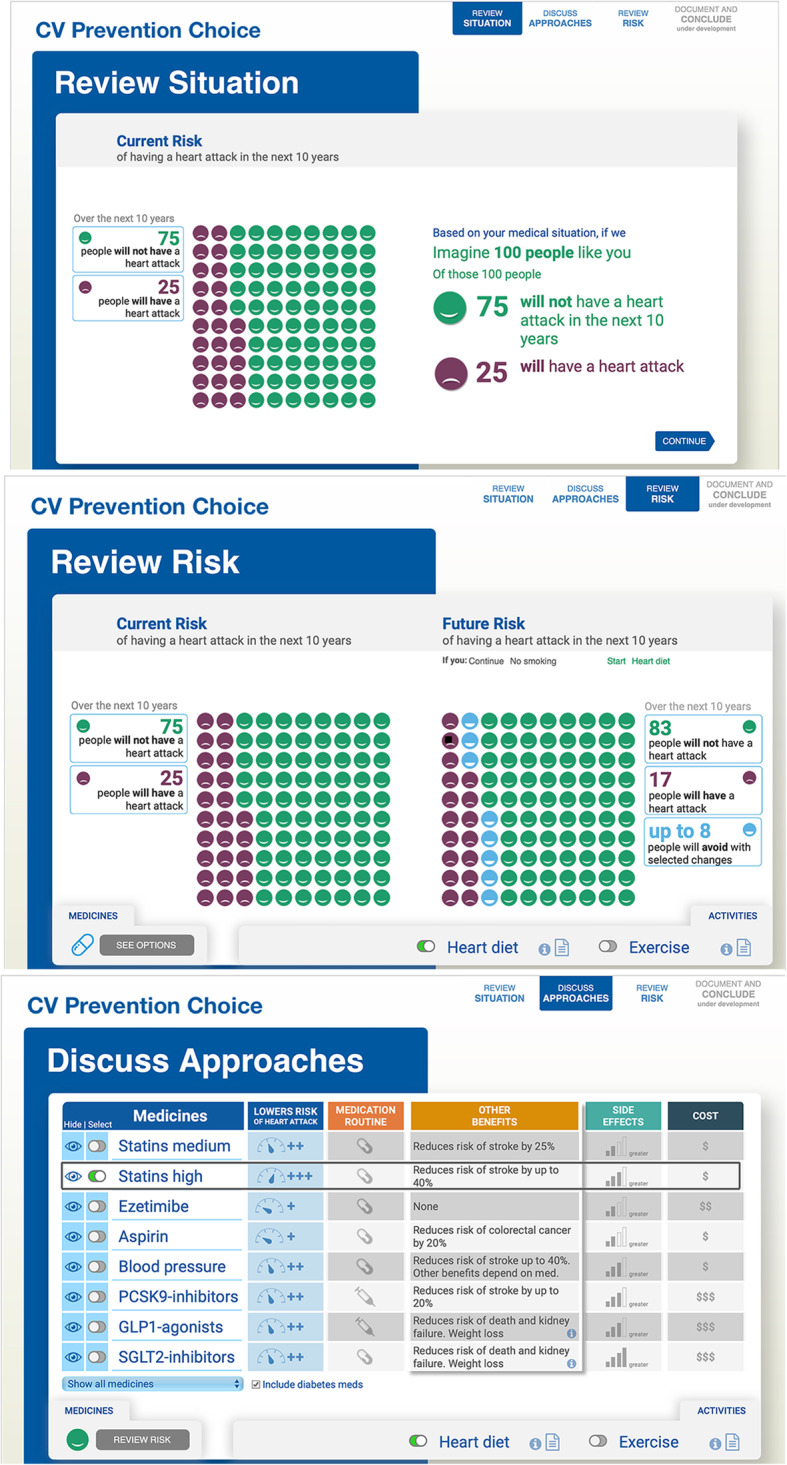
CV Prevention Choice

CV Prevention Choice (Fig. 1) extends Statin Choice by including other lifestyle and pharmacological interventions in the model of risk reduction. CV Prevention Choice considers the risk-reducing effects of the interventions discussed in the ACC/AHA 2019 Primary CV Prevention Guidelines including smoking cessation, adoption of the Mediterranean diet, vigorous exercise, and preventive medications. In addition to statins, the tool includes information about other cholesterol-lowering drugs (e.g., statins, ezetimibe, PCSK9-inhibitors), blood pressure lowering medications, aspirin, SLGT-2 inhibitors, and GLP-1 agonists.

Other tools use the epidemiological association between risk factors and estimated ASCVD risk to model what-if scenarios and use these models in patient counseling. CV Prevention Choice uses a different approach. First, like Statin Choice, it uses, when available, the relative risk reduction estimates from RCTs to model the effect of interventions on CV risk. Second, it supports the co-creation of a plan of care that may include lifestyle intervention and medications, and only then, it displays the effect of this plan on the patient’s CV risk. The process can be repeated until the patient and the clinician arrive at a feasible and sensible plan that achieves a desirable reduction in the estimated risk. The tool ends by aiding in the production of a patient handout and text that the clinician can add to the encounter notes; this text includes the patient’s risk, the preventive regimen agreed upon, and the expected risk reduction associated with it.

CV Prevention Choice was iteratively refined and field tested within actual clinical encounters, using procedures we have employed in other studies [31, 32]. CV preventive care encounters were video-recorded and reviewed by members of the study team with expertise in CV prevention and human-centered design. Refinements were made in response to researcher observed misuses, inaccurate presentations, or poor usability experiences, as well as in response to clinician recommendations for adding, editing, or removing content to improve the design, content, or usability. The final tool was prepared as a web application for integration into the EPIC electronic medical record system.

### Setting

This study will take place in three health systems in the Mayo Clinic Care Network (MCCN). The MCCN is a collaborative network linking more than 40 health systems around the globe with the research, education, and clinical expertise of Mayo Clinic. MCCN health systems have been involved in a variety of implementation-focused research studies and as such have served as a laboratory for exploring the challenges and opportunities of translating research into practice [22, 33, 34].

### Study design

**Fig. 2:**
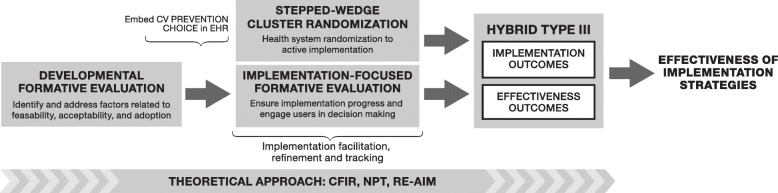
Study design

This study will employ formative evaluation methods [35] and a Hybrid Type III [36] stepped wedge cluster randomized design, as shown in Fig. 2. The stepped wedge approach allows for each system to be exposed to the intervention, while the hybrid design with both implementation and effectiveness outcomes appreciates the consistent evidence base for SDM tools while addressing the more limited evidence on successful implementation strategies for bolstering SDM uptake and use.

**Fig. 3:**
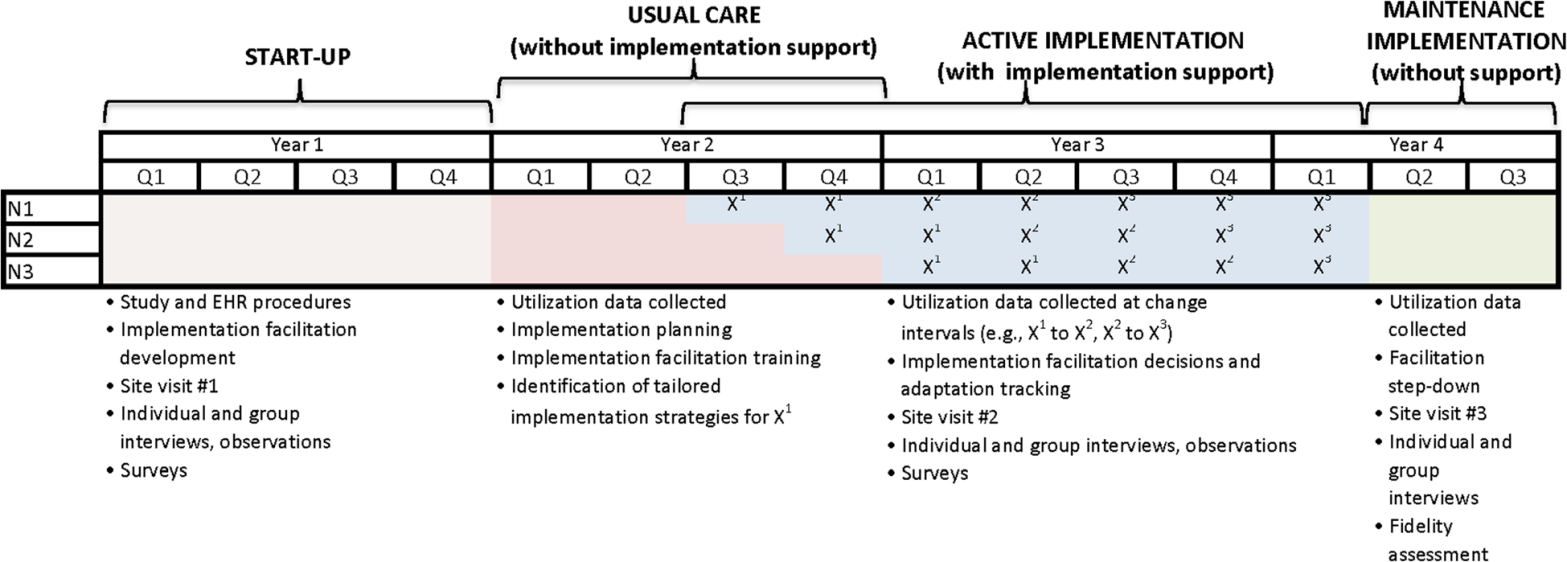
Study schema by quarter

The stepped wedge design will be implemented across the three health systems, indicated in Fig. 3 by N1, N2, and N3, with the start of the active implementation stage determined by study randomization. All health systems will have a minimum of two quarters (6 months) in usual care. During this stage, CV Prevention Choice will be available in the EHR, but no “active” notification or in-depth clinician training will take place, mimicking the conditions under which many decision support tools exist, i.e., available in the EHR without support or integration into clinical workflow.

Health systems will have a minimum of five quarters (15 months) of active implementation. This stage will involve implementation-focused formative evaluation and implementation facilitation. Implementation strategies will be systematically tracked and refined or adapted at 6-month intervals, portrayed as dose changes in Fig. 3. The final two quarters (6 months) of maintenance implementation mark step down of active implementation activities and the start of final data analysis. During this stage, we will evaluate normalization (i.e., routine use) of CV Prevention Choice in practice.

### Theoretical approach

The study employs three theoretical approaches: the Consolidated Framework for Implementation Research (CFIR) [37], the Normalization Process Theory (NPT) [38, 39], and the RE-AIM evaluation framework [40, 41]. CFIR is a determinant framework [42] with constructs in five domains (the intervention, inner and outer setting, the individuals involved, and the process by which implementation is accomplished) [37]. CFIR constructs are well-suited to call attention to the factors that might affect implementation in this study, including clinician attitudes, leadership engagement, and organizational policies or payment structures. CFIR will be used in the formative evaluation, in the development of data collection instruments, and in data analysis, as well as in the process of identifying and tailoring implementation strategies.

NPT is an implementation theory that guides explanation or understanding of causal influences on implementation outcomes. Where CFIR draws attention to categories of determinants that affect implementation outcomes, NPT focuses “attention on the processes by which complex interventions are made workable and integrated in everyday practice” [38]. NPT constructs will be assessed using surveys and qualitative data collection with clinicians, providing a way to assess mechanisms of routinizing CV Prevention Choice in the various participating health system practices.

Finally, the RE-AIM framework will be employed to evaluate implementation and intervention outcomes across its five dimensions: reach, effectiveness, adoption, implementation, and maintenance. This framework will be used in the planning stage and during iterative implementation strategy adaptation, in addition to its use in the evaluation of summative study outcomes [40]. Each construct of the RE-AIM framework will be assessed using a combination of EHR data, surveys, individual or group interviews, and video observation. We will also assess the relative impact of implementation strategies on these constructs.

### Implementation facilitation

The primary implementation strategy in this study is implementation facilitation, which engages stakeholders to develop and problem solve implementation strategies based on local context [43]. Facilitation has been successfully employed in a range of primary care settings to increase adoption of evidence-based practices [44] and improve chronic disease care measures [45]. For this study, each health system will identify an implementation facilitator who will lead efforts with their sites. This internal facilitator will help make decisions about what implementation strategies to deploy, with support from the study team and after review of local data, e.g., metrics on tool use. He or she will also convene the local implementation team that will assist with implementation in the participating sites (e.g., administrative and IT personnel). The principal investigator in each health system will serve the role of clinical champion. One of the study investigators will serve as an external implementation facilitator, whose job will be to support the work of the internal facilitators.

During active implementation, the implementation team will assess implementation progress (e.g., qualitative assessments of challenges) and outcomes (e.g., metrics of tool adoption). The CFIR framework will be employed to maintain focus on the various determinants of implementation success. Implementation tools such as the CFIR-ERIC Implementation Strategy Matching Tool (© 2019 The Consolidated Framework for Implementation Research), as well as other taxonomies of strategies [46, 47], will be used to facilitate conversations with implementation teams and guide decisions based on systematic assessment of needs, including recognized barriers to SDM uptake [48]. We will also engage stakeholders in a collaborative process, similar to the intervention mapping or concept mapping approaches described by others, to connect identified barriers to appropriate strategies [49, 50]. To allow adequate time to develop, deploy, and assess strategies, the implementation teams will convene to review local data and take actions (including adoption, de-adoption, and maintenance of individual strategies) at approximately 6-month intervals.

The implementation facilitators will track decisions and adaptations in implementation logs in order to understand their contributions to implementation success [51, 52]. These logs will serve to estimate the bundles of strategies implemented in each interval (i.e., the “dose” of implementation effort) [52, 53]. Implementation teams will also engage in periodic reflections [54] which involve systematic documentation of regularly scheduled guided discussions about implementation (e.g., activities, challenges, and adaptations).

### Data collection

Surveys will be administered via Qualtrics, a HIPAA-compliant electronic survey system, to eligible individuals. Participants will be emailed an initial invitation to complete the survey online, as well as up to one follow-up notification, using the Qualtrics survey administration tool (Qualtrics, Provo, UT). If a participant does not respond within 10 days of the second notification, a paper version of the survey will be provided.

Participants will be invited in-person or by email to individual and group interviews, which will be conducted in-person or by phone/video conference by a trained member of the study team. Interviews will be recorded with permission and transcribed verbatim for analysis. All data will be stored on password-protected servers. The study team will receive all EHR data monthly via a secure electronic data transfer stored on a secure server.

### Outcomes and measures

Table 1 summarizes the study process and schedule of data collection for implementation outcomes (Aim 1). First, surveys will be administered to clinicians and stakeholders during the start-up phase. Clinician and stakeholder startup surveys will assess organizational readiness using the Organizational Readiness to Implement Change (ORIC) instrument [55], feasibility using the Feasibility of Intervention Measure subscale of the AIM-IAM-FIM measure [56], and items related to attitudes toward the EHR. The clinician version will also assess participants’ attitudes toward SDM in relation to time constraints, liability, conflict with clinical judgment or guideline recommendations, and decisional responsibility [57]. Focus groups and interviews with clinicians and stakeholders will further assess attitudes and implementation context. Observations and document review will be used to characterize the implementation context and map existing clinic workflow.

**Table 1:** Study process schedule

	Enrollment of health systems/sites	Usual care	Active	Maintenance
**Time period**	-t1	t0	t1	t2
**Enrollment:**				
Clinician consent	X			
Patient consent				X
**Intervention**				
CV Prevention Choice in the EHR		X	X	X
Training of internal facilitators		X		
Identify implementation strategies		X		
Implement identified strategies			X	
Monitor implementation strategies and dose change as applicable			X	
**Evaluations**				
Site visit	X		X	X
Observations	X		X	
Video-recorded encounters				X
**Assessments**				
Utilization collection		X	X	X
Interview stakeholders	X		X	
Interview clinicians	X		X	X
Stakeholder survey	X			
Attitudes toward EHR	X			
Organizational Readiness to Change	X			
Implementation feasibility	X			
Clinician Survey	X		X	
Attitudes about SDM	X		X	
Attitudes towards EHR	X			
Organizational Readiness to Change	X			
Implementation feasibility	X			
Impressions and use of CV Prevention Choice			X	
SDM Normalization (NOMAD Scale)			X	
Patient Survey				X
CARE Measure				X
SDMQ-9 Scale				X

-t1 – Completed within the quarter prior to CV Prevention Choice being available in the EHR

t0 – From the time CV Prevention Choice is the EHR until the system cross-over to active implementation per the randomization scheme (2–4 quarters after starting)

t1 – Starts at time of crossover (5–7 quarters after crossover)

t2 – At the end of active implementation, the maintenance phase starts and will last for two quarters

Clinicians will be surveyed again at approximately the midpoint of the active implementation stage. The survey will repeat items on SDM attitudes and acceptability. It will also include items related to CV Prevention Choice impressions and use, as well as items from the NoMAD questionnaire, which assesses how well SDM has been normalized into routine clinical practice [58–60]. NoMAD questions are grouped in domains representing the constructs of NPT, which together provide a framework for understanding how an intervention is implemented, embedded, and integrated into practice.

Over a 3-day period during the maintenance stage, a convenience sample of encounters will be randomly selected for video-/audio-recording to assess SDM fidelity. Patients in those encounters will be asked to complete a survey that includes the Consultation and Relational Empathy (CARE) Measure to assess the level of patient centered care [61], as well as the 9-item Shared Decision Making Questionnaire (SDMQ-9), which assesses the extent to which the patient felt involved in the decision-making process [62].

These implementation outcomes and measures are summarized using the RE-AIM framework in Table 2. REACH is a measure of how many individuals were willing to participate. In this study—because CV Prevention Choice will be available to all site clinicians—we define REACH as the proportion of all eligible providers who activate the link to it at least one time. This is a proxy for willingness to participate (as distinct from broader adoption and use). For each clinician, we will collect practice type (e.g., family medicine, internal medicine) and clinician job classification (e.g., MD, NP) to assess differences between participants and non-participants.

**Table 2:** RE-AIM implementation outcomes

Construct	Data source	Measure
Reach	• EHR data	• Proportion of clinicians who accessed CV Prevention Choice • Characteristics of participants versus non-participants
Effectiveness	• Clinician survey • Clinician interviews	• Survey of attitudes about SDM [40] • Perceptions of effectiveness of CV Prevention Choice, and unintended consequences of its use
Adoption	• EHR data • Clinician interviews	• Proportion of eligible clinicians who used CV Prevention Choice • Factors affecting adoption of CV Prevention Choice and implementation strategies
Implementation (fidelity and challenges)	• Video-recorded encounters • Post-encounter patient surveys • Clinician and stakeholder interviews • Periodic reflections and strategy tracking/change logs • Observations and workflow mapping	• Fidelity checklist applied to video-recorded clinical encounters [25] • CARE Measure and Shared Decision Making Questionnaire-9 [61, 62] • Self-reported assessments of implementation challenges and adaptations to delivery
Maintenance and normalization	• EHR data • Clinician survey	• CV Prevention Choice use at start and end of implementation step-down stage • Repeated CV Prevention Choice use with the same patients • NoMAD survey [58–60]

As an implementation outcome, EFFECTIVENESS will be assessed qualitatively and will include clinicians’ perceptions not only of how well the CV Prevention Choice tool works, but of any unintended consequences of its use. These perspectives will be assessed during individual and group interviews, using a semi-structured interview guide.

ADOPTION is a primary outcome measure that will be used to assess whether CV Prevention Choice is being used in clinical visits. We will use data from the EHR to determine the proportion of eligible clinical visits in which CV Prevention Choice was recorded as being used. Individual and group interviews will further elicit perceptions of the factors related to clinician adoption.

IMPLEMENTATION will be assessed using a fidelity checklist with a sample of video-recorded clinic visits. Study staff will audio-video record (with clinician and patient consent) eligible encounters taking place during a randomly selected 3-day period around the transition from the active to maintenance implementation stage. Patients from those encounters will be asked to complete the survey. Individual and group interviews with clinicians will also be used to capture implementation challenges and self-reported adaptations. The implementation team’s periodic reflections, observations, and strategy tracking/change logs will also be collected for analysis of implementation challenges and reasons for strategy deployment and refinement.

MAINTENANCE will be assessed by determining CV Prevention Choice use at the end of the maintenance implementation period for each cluster. We will also assess normalization of SDM in practice using NoMAD.

The effectiveness outcome for this trial (Aim 2) estimates the extent to which CV Prevention Choice results in individual CV prevention plans that are evidence-based, congruent with each person’s estimated CV risk, and feasible within each patient’s personal and psychosocial context. While we expect variability in uptake of preventive interventions as a reflection of patient preferences, i.e., warranted variation, we hypothesize that individuals at lower baseline risk, on average, will be less likely to take up preventive interventions than individuals at higher risk. This is because low-risk individuals at baseline (e.g., in middle-aged women with high HDL cholesterol levels) or after intervention (e.g., a 65-year-old active man with well-controlled hypertension and diabetes on high-dose statins, an ACE inhibitor, and an SGLT-2 inhibitor) may find the incremental benefit from adding a preventive intervention at this point to be associated with a very small beneficial risk reduction that many, but not all, participants would find not worth the potential harms and definite burdens and costs. At higher baseline risks, more interventions remain desirable despite their potential harms, costs, and burden. Although the interventions patients can consider include lifestyle and pharmacological interventions, in this pragmatic trial, only the latter can be feasibly ascertained using the EHR. Therefore, the main effectiveness variable will be an assessment of whether medication usage is congruent with 10-year CV risk. This limitation is justified by (a) the pragmatic need to impose minimal burden on practices and participants, (b) the secondary nature of these outcomes in this hybrid trial, and (c) the limitations of most approaches (e.g., self-report, pill-counting, refill pharmacy profiles) to ascertain treatment adherence to preventive interventions.

### Eligibility criteria

#### Health systems

Health system criteria for participation included membership in the MCCN, as well as commitment to recruit at least four practices (e.g., family medicine, women’s health, preventive cardiology) with at least five clinicians each who see adult patients 40–75 for preventive care; provide prioritized information technology (IT) support to integrate and maintain CV Prevention Choice within the EPIC EHR and implement data abstraction procedures; and identify staff to champion the study, lead implementation facilitation activities, and support primary data collection (e.g., surveys and interviews) at the practice sites. Practice size minimums were meant to ensure adequate numbers of clinicians and patients for a study with implementation and effectiveness outcomes.

#### Clinicians and stakeholders

Clinicians will be eligible if they are affiliated with the participating health system practices and see patients ages 40–75 for preventive care. Health system stakeholders will be eligible if they are involved in the administrative, research, quality improvement, or IT systems related to implementation of CV Prevention Choice.

#### Patients

Patients will be selected following the 2019 ACC/AHA guidelines for CV primary prevention [4]. Patients without a first atherothrombotic clinical event, between 40 and 75 years of age, with or without diabetes, who receive preventive care in included practices during the course of the study, are eligible to participate. We anticipate that some otherwise eligible patients will not participate in preventive care because of advanced disease, limited life expectancy, or any other condition that makes focusing on CV prevention inappropriate. As part of the implementation outcomes, we will monitor for the exclusion of persons from SDM for any reason, including insufficient vision or cognition.

### Recruitment and ethical procedures

This study has been approved by the Mayo Clinic Institutional Review Board (20-002772), which is the IRB of record for this study. Consistent with the pragmatic nature of this trial, encounter-level data to assess study outcomes will be abstracted for all eligible clinicians and patients with eligible visits in the affiliated health system sites during the study period. These data will be abstracted for research purposes and direct identifiers of individuals will be removed (names, dates, and emails). We will follow any local policies for data authorization.

Individual recruitment and consent procedures will be followed for surveys, interviews, and observations. All eligible clinicians and stakeholders will be recruited and consented to complete surveys and participate in qualitative individual or group interviews. Recruitment for those activities will take place at site meetings and individually in-person or through email from members of the study team. Oral consent scripts will be used for interviews and surveys will include consent language. For the audiovisual recording of clinical encounters to assess fidelity, study personnel will assess eligibility and notify participating clinicians of eligible patients with upcoming appointments. Clinicians who agree to participate will complete written informed consent. Patients will be approached prior to the encounter, and those who agree will be recruited and complete written informed consent. Clinicians, stakeholders, and patients will be informed of the voluntary nature of these activities, as well as the protections for data privacy. Because this trial assesses an educational tool, a data monitoring committee is not required, nor will one be implemented. This study will employ a data safety and monitoring plan to guide our efforts to monitor participant safety, data completeness, and adherence to study protocol.

### Data analysis plan

#### Randomization

The study statistician will create a simple randomization scheme to randomly assign the order of crossover to active implementation for the three health systems. This information will be made available to study staff at the start of the usual care stage (for planning purposes). Health system staff and clinicians will not be blinded to the stage of the intervention nor any study staff. Patients recruited for fidelity assessment will be made aware of the intervention as an educational tool.

#### Implementation outcome analysis

##### Quantitative analyses

For implementation REACH, we will calculate the proportion of clinicians who activated the tool link (from among clinicians who had an eligible visit during that period). Participation rates will be compared by practice type and job classification. For ADOPTION, we will calculate the proportion of eligible clinical visits at each site where tool use was documented in the EHR; the difference in adoption at the midpoint of usual care and at every 6 months and at the end of the active implementation stage will reflect the difference between simply embedding the tool in the EHR and full implementation of tailored strategies. As a proxy for clinicians’ long-term use and perceived value (MAINTENANCE), we will evaluate the proportion of eligible visits during which CV Prevention Choice was used repeatedly with the same patient. For IMPLEMENTATION (fidelity), two trained members of the study team will work in duplicate to review recordings against a fidelity checklist [25]. In any cases where there is lack of concordance, a third trained member of the study team will arbitrate. Patient post-encounter surveys will be analyzed descriptively by site and system to understand fidelity to components of SDM conversations [63].

The NoMAD survey will be analyzed at the system level. Other secondary analyses (e.g., by site) will be completed as appropriate based on response. Response rates and unadjusted average estimates along with standard deviations for each system will be presented. Considering that clinicians within a healthcare system may have similar responses to the NoMAD questionnaire, a clustering effect due to proximity, shared administration, and similar exposure to adopted active implementation strategies could occur. The analysis will account for this clustering effect. NoMAD is a continuous outcome converted to a 0-100-point scale in which the adjusted average effect of system will be modeled at the maintenance implementation time point adjusting for the initial response in the active implementation phase. We will construct a hierarchical model with fixed effects of initial NoMAD score and any system covariates and the random effect of site and system. The model will produce an estimated effect along with a 95% confidence interval for each system.

##### Qualitative analyses

Interview transcripts, field notes, documents collected during observations, and notes from periodic reflections will be subject to directed content analysis by at least two team members [64, 65]. Directed content analysis takes into consideration the existing literature and theories or frameworks that informed the research. In this study, coding of content in the data will start with the domains and related constructs from CFIR. Computer-assisted qualitative data analysis software (NVivo 12.3., QSR International) will facilitate data organization and analysis, including analytic memo writing that summarizes impressions and explores and documents connections in the data. We will further use side-by-side comparisons of qualitative and quantitative findings on common constructs (e.g., attitudes toward SDM) using RE-AIM QuEST [66].

#### Effectiveness outcome analysis

For each evaluable encounter, the outcome of interest will be coded as a binary outcome indicating whether any medication among the relevant set was prescribed. This outcome will be assessed using a risk-adjusted mixed logistic regression model. The model will adjust for each patient’s CV risk score—akin to adjusting the model for case-mix taking into account the risk of death to appropriately estimate differences found assessing quality of hospitals on mortality. The model will account for the potential clustering effect that would be expected among patients along with clinician and practice within the health system (random effect, to account for correlation of patients with multiple encounter, patients seen by the clinician and clinicians within practices, with the inclusion of practices as there could be variation among sites within a system), roll-out time period (fixed effect, to adjust for confounding of intervention with time trends), and tool usage variable (fixed effect, usual care vs CV Prevention Choice, the effect of interest) [67]. Patient and site covariates will be included to adjust for differences in patients and sites across units and over time. Model assumptions will be verified and findings will be reported according to best practices including CONSORT guidelines on cluster randomized trials [68]. In secondary analyses, the models will be extended using interaction terms to examine several types of heterogeneity of treatment effect (HTE) due to possible changes or improvements in effectiveness of the intervention (interaction of intervention with time period) [48]. One secondary analysis is the interaction between tool use and the time interval (starting at passive implementation in EHR and increased by quarter until the start of maintenance, to assess if there are different effects of usage depending on the time point). Although we do not hypothesize difference by sex or race, additional HTE to be evaluated include potential interaction effects among patient sex and patient race (minority vs. not) [69]. These will be reported in study findings as appropriate.

#### Sample size and power

For the implementation outcomes, the number of clinicians or other stakeholders we expect to enroll is based on the minimum numbers of clinicians in the participating sites. We anticipate a minimum of 60 clinicians and 20 stakeholders eligible to participate in interviews and surveys. While sample sizes for qualitative inquiry are not dictated by statistical power analyses, the expected numbers of individual or group interviews are well within the parameters for qualitative content analysis and should be sufficient for qualitative assessment of implementation factors [70, 71]. Similarly, survey analysis will be undertaken with appropriate statistical considerations in mind. Any limitations will be reported alongside results as appropriate.

For fidelity assessment, we anticipate approximately 10 eligible encounters per day (per system/site). Our experience in similar studies [72–74] suggests we will be able to recruit and enroll in about one third of eligible encounters. This will yield approximately 27 to 36 audio-/video-recorded files for analysis across the three health systems. In this study, the purpose of the fidelity checks is to understand how providers are using CV Prevention Choice, rather than to assess differences between intervention and usual care groups. Thus, statistical power considerations are not applicable.

For the effectiveness outcome, we estimated the intervention effect that can be detected with our stepped wedge design on proportion of patients with a medication of interest on record to be detectable at a 10% increase over usual care with CV Prevention Choice. We assumed the following: (1) there will be > 400 evaluable patients per rollout in a health system during the study period (i.e., 1600 patients per health system with a total of 4800 encounters evaluable), (2) the intracluster correlation (ICC) is 0.10 with a between cluster variations of 0.025, and (3) a two-sided level of 0.05 for significance tests. Patient estimates are based on previous work to implement statin choice, where we saw a mean of 300 uses per month in each of three health systems [22]. Using methods for estimating sample size in a stepped wedge RCT [75], we estimate 97% power to detect the 10% increase in medications being recorded/prescribed in the EHR.

#### Trial oversight and dissemination of findings

A trial steering committee, comprised of the principal investigators and co-investigators, will support overall study conduct, including review of the data safety and monitoring plan. The steering committee will also form a publication plan for dissemination of study findings. Any important modifications to the protocol (e.g., eligibility criteria) will be approved by the steering committee and the IRB before use. Institutional IRB policies require that participants be notified of some protocol changes, e.g., changes in study risk. These are not anticipated with the educational nature of this study.

## Discussion

While evidence-based, adoption of SDM in CV preventive care is currently not widespread. This study leverages the team’s expertise in SDM, practice-based research, and implementation and evaluation science in real-world settings. It starts where prior efforts have stopped; it makes EHR deployment a baseline achievement so that the focus is on testing the effects of active, theory-guided, implementation strategies to achieve RE-AIM outcomes and normalization of SDM in routine care.

The strengths of this study include (1) its systematic but flexible approach to tracking implementation strategies in real-world practice and at intervals that allow for identification of strategies that made a difference, (2) the inclusion of an implementation step-down period for assessing SDM after implementation support is removed, (3) the availability of EHR functionality to assess adoption and use, and (4) the use of theory-driven mixed methods. The participation of three health systems will allow evaluation in varying organizational and geographic contexts and with diverse patient populations. Limitations include the relatively small number of health systems involved, and the restricted data set available in the EHR for the assessment of effectiveness outcomes.

## Conclusion

Effective, risk-concordant, and well-implemented preventive care in clinical practice is paramount to mitigate the effect that CV disease has on public and personal health. Furthermore, clinical interventions should be congruent with patient values, goals, and priorities. SDM is a method of care that should improve the quality of CV prevention in clinical practice. If successful, the proposed study should contribute to the evidence base about the relative contributions of different implementation strategies in real-world practices, and about the relative contribution of an implementation of SDM—using an encounter SDM tool embedded in the EHR—to primary CV prevention. This evidence should, in turn, inform practice guidelines and other policies seeking to reduce the burden of CV disease by promoting the use of SDM to shape preventive regimens that make sense in the lives of at-risk patients.

## Data Availability

The full protocol and datasets used and/or analyzed during the current study may be made available from the corresponding author on reasonable request after one year from the final publication on the primary aims and with appropriate resources.

## Abbreviations

ASCVD: Atherosclerotic cardiovascular disease
CARE: Consultation and relational empathy
CFIR: Consolidated Framework for Implementation Research
CV: Cardiovascular
EIF: External implementation facilitator
EHR: Electronic health record
IIF: Internal implementation facilitator
MCCN: Mayo Clinic Care Network
NPT: Normalization Process Theory
ORIC: Organizational Readiness to Implement Change
SDM: Shared decision making
